# Extending the DSC paradigm: some areas for future research

**DOI:** 10.3389/fpsyg.2014.00889

**Published:** 2014-08-26

**Authors:** John L. Protevi

**Affiliations:** French Studies, Louisiana State UniversityBaton Rouge, LA, USA

**Keywords:** durkheim, tarde, deep social cognition, quorum sensing, enaction, mind-in-life continuity

John Stewart proposes we study what we can call “deep social cognition” (DSC), as opposed to the mere embedding or extending or modifying of cognition by social factors, as Stewart characterizes the tradition of “social cognition” studies to date.

DSC claims that for humans our basic or non-empirical categories—space, time, identity, equality, and so on—are relative to social practices. One could say that DSC takes up the mind-in-life continuity thesis (Thompson, 2007) and explores it relative to human cognition. To fight the representation lists, early enactivists insisted that whatever the content of cognitive processes enacted in the co-constitution of organismic value and environmental affordance, those contents were in fact enacted and not objective reflections (realism) or subjective creations (idealism).

A common enactivist strategy here was to study single-celled organisms (e.g., *E. Coli*). If they displayed cognition qua sense-making, then the ground floor of the mind-in-life continuity thesis would be established and it would then be a matter of studying qualitative shifts in the continuum of organismically rooted cognition: consciousness vs. sentience, self-consciousness vs. “mere” consciousness, etc. Once the baseline is established, however, Stewart implies, there has to be a follow up investigation of the correlation in human beings of historical/social forms of life and basic categories.

After this mise-en-scène, in the remainder of the comment I will raise some points not so much in criticism as in hopes of offering further research avenues.

1. Might gene expression regulation in populations of bacteria allow us to think the evolutionary depth origins of DSC? For an introduction to the issue of bacterial quorum sensing, see Joint et al. (2007). For an ambitious attempt at articulating the “origins of sociable life,” see Hyrd (2009).
2. DSC falls in the tradition of naturalizing Kant. A figure of note here is F. A. Lange, author of an influential *History of Materialism* (1974) [1866]. Lange adds an evolutionary and socializing perspective to the naturalizing of Kant begun by Helmholtz in an individualist and representationalist frame (Hatfield, 2012). For Lange, however, the conditions of possibility of experience are species-specific adaptations. Lange in turn influenced Nietzsche's position that affective-cognitive patterns are relative to “forms of life” (Stack, 1991; Cox, 1999). Finally, there is Welshon (2014), which claims Nietzsche as precursor to “dynamic embodied-embedded cognitive science.” It would thus be very interesting for the enactivist community to follow up on possible DSC—Nietzsche connections.
3. Stewart's use of Durkheim's top-down model could be complemented by the bottom-up methodology of his great rival, Gabriel Tarde. Latour (2002) can serve as an introduction to Tarde; see De Jaegher (2013) for a recent enactivist piece thematizing top-down / bottom up complementarity in social life. Tarde criticizes Durkheim for giving himself his “social facts” as already established: in this case, the categories of time, space, subject, object, etc as reflecting social forms. Tarde insists, however, on an account of the genesis of such categories from a molecular field of differences. Tarde is not really an individualist, however, as the basic social units are not really units at all, but “monads” in a constant state of variation and imitation of others. For Tarde, then, the big universals—social forms, basic categories—are formed and held together by minute “repetitions with a difference” (to adopt the terms of Gilles Deleuze). So Tarde insists students of society need a bottom-up methodology—though of course once the categories are in place they guide the socialization of thought in succeeding generations, so there is room for top-down effects as well.
  Tarde insists however that the social facts are fragile and in need of constant reinforcing—just how much innovation is allowed before top-down enforcement squelches them, or indeed, before they take hold and change the top-level structures? So adding a bottom-up Tardean perspective allows us to account for different rhythms of change in categories in a way that Durkheim's progressive model doesn't (as I understand it, Durkheim has an account of modernity as increasing specialization in the division of labor). Hence Tarde's critique of Durkheim:
  Mr. Durkheim spares us such terrible tableaux. With him, no wars, no massacres, no brutal invasions. Reading him, it seems that the river of progress has flowed smoothly over a mossy bed undisturbed by froth or somersaults. […] Evidently, he inclines towards a Neptunian, rather than a Vulcanian, view of history: everywhere he sees sedimentary formations, nowhere igneous upheavals. He leaves no place for the accidental, the irrational, this grimacing face at the heart of things, not even for the accident of genius. Latour et al. (2008).
4. On the general point of a historical/social genesis of basic categories, the recent “ontological turn” in anthropology springs to mind. The main references here are Viveiros de Castro (2009) and Descola (2005). A complex movement, bound up with strong debates on cultural relativism inside and outside anthropology, I mention it here simply for the sake of connecting Stewart's DSC project with other social science movements. A brief extract from Viveiros de Castro et al. (2014) will show its relevance: “the anthropological concept of ontology [entails] the multiplicity of forms of existence enacted in concrete practices, where politics becomes the non-skeptical elicitation of this manifold of potentials for *how things could be* ….”
5. Finally, Sohn-Rethel is a complex and difficult thinker with whom I am not really familiar. Consequently, I will restrict myself here to a reference to Read (2014), with the remark that Read also looks to the Italian Autonomia thinkers, in particular Virno's reading of Marx on the “general intellect” as it relates to the post-industrial economy. This field of thought bears on Stewart's discussion of financial capitalism, which cannot be underestimated as a vitally important philosophical/political topic, just as nuclear power and global climate change rose to the forefront of thought in the eras in which they assumed dangerous potentials.

## Conflict of interest statement

The author declares that the research was conducted in the absence of any commercial or financial relationships that could be construed as a potential conflict of interest.
